# The Medical Society of Montgomery County, Tenn.

**Published:** 1850-02

**Authors:** 


THE MEDICAL SOCIETY OF MONTGOMERY COUNTY, TENN.

We received, some weeks ago, an excellent paper, read before the above Society, by Dr. E. B. Haskins, "On the Diagnostic Value of the Urine." We hoped, at one time, to publish it in this number, but were compelled to postpone the publication, on account of matters of a more pressing character. The essay will appear in the March number, and will be read with interest.

